# Concomitant Intubation with Minimal Cuffed Tube and Rigid Bronchoscopy for Severe Tracheo-Carinal Obstruction

**DOI:** 10.3390/jcm12165258

**Published:** 2023-08-12

**Authors:** Jacopo Vannucci, Rosanna Capozzi, Damiano Vinci, Silvia Ceccarelli, Rossella Potenza, Elisa Scarnecchia, Emilio Spinosa, Mara Romito, Antonio Giulio Napolitano, Francesco Puma

**Affiliations:** 1 Department of Thoracic Surgery and Lung Transplantation, University of Rome Sapienza, Policlinico Umberto I, 00161 Rome, Italy; 2 Department of Thoracic Surgery, University of Perugia Medical School, Ospedale Santa Maria della Misericordia, 06134 Perugia, Italy; rosannacapozzi@hotmail.it (R.C.); vinci.damiano@libero.it (D.V.); silvia1.ceccarelli@ospedale.perugia.it (S.C.); rossella.potenza1987@gmail.com (R.P.); elisascarnecchia@tiscali.it (E.S.); emiliospinosawmb@gmail.com (E.S.); mara.romito@ospedale.perugia.it (M.R.); antongiulionapolitano@gmail.com (A.G.N.); fr_puma@yahoo.it (F.P.)

**Keywords:** tracheal stenosis, rigid bronchoscopy, airway, anesthesia, intubation

## Abstract

Background: Our aim was to report on the use of an innovative technique for airway management utilizing a small diameter, short-cuffed, long orotracheal tube for assisting operative rigid bronchoscopy in critical airway obstruction. Methods: We retrospectively reviewed the clinical data of 36 patients with life-threatening critical airway stenosis submitted for rigid bronchoscopy between January 2008 and July 2021. The supporting ventilatory tube, part of the Translaryngeal Tracheostomy KIT (Fantoni method), was utilized in tandem with the rigid bronchoscope during endoscopic airway reopening. Results: Indications for collateral intubation were either tumors of the trachea with near-total airway obstruction (13), or tumors of the main carina with total obstruction of one main bronchus and possible contralateral involvement (23). Preliminary dilation was necessary before tube placement in only 2/13 patients with tracheal-obstructing tumors (15.4%). No postoperative complications were reported. There was one case of an intraoperative cuff tear, with no further technical problems. Conclusions: In our experience, this innovative method proved to be safe, allowing for continuous airway control. It enabled anesthesia inhalation, use of neuromuscular blockage and reliable end-tidal CO_2_ monitoring, along with protection of the distal airway from blood flooding. The shorter time of the procedure was due to the lack of need for pauses to ventilate the patient.

## 1. Introduction

Rigid bronchoscopy (RB) is an effective treatment for the palliative care of tracheobronchial obstruction and, in selected cases, may have a role in preparation for curative resection [1,2,3,4,5]. The management of ventilation during RB is crucial. This is because the rigid bronchoscope can only partially replace the orotracheal tube. Its employment is generally performed in patients with critically compromised airways. Using the current techniques, oxygenation and ventilation are achieved through the lumen of the rigid bronchoscope, which is uncuffed and open during the interventional maneuvers. The ventilator circuit thus results in considerable leaks of the air volume around and across the instrument. Since anesthesiologists and surgeons have to share the airway control during RB, they need to optimize the coordination of the ventilation and apneic periods, along with any operative and non-operative phases [6,7]. At highly dedicated institutions, where all members are well-trained and expert, complication rates and procedural difficulties are minimized, but some cases require more than good experience and expertise considering the extreme of asphyxia and maximal airway obstruction.

Historically, RB has been developed for different purposes. Although flexible endoscopes have been successfully developed with increasing technology and availability, RB remains important in the surgical armamentarium for different reasons. Rigid instruments have proven to be effective in debulking obstructive endoluminal tumors and have not been surpassed by a more recent alternative in this setting. In airway disease, anesthesia is demanding considering that patients have a critical ventilation impairment with a large variety of respiratory distresses or dyspnea. The first goal that the involved crew needs to achieve is a deal on how to maintain a stable oxygen level throughout the entire procedure without hampering the operative phase. Interventional techniques on the affected upper airway are increasing, and difficult airway management is not a rare event. Many technologies are under development to offer patients better solutions. Lasers and medical devices are currently expanding indications, capacity, and biocompatibility, and the applied endoscopic procedure consequently needs further development itself. As said before, the severe limitation of sharing the airway for the necessary ventilation of the patient and the lumen patency retrieval is a tangible point of progress to be implemented. An expert dedicated team is essential in managing patients with severe tracheal or carinal obstructions presenting respiratory distress [8].

Different ventilation techniques can be used and the choice for the best one is often dependent on the anesthesiologist’s experience, the procedure to be performed and the specific situation, as none of the described four ventilation techniques have a demonstrated superiority over the others [8]. Regardless of the method of ventilation employed, intraoperative airway management can be challenging in two specific situations, such as severe tracheal and carinal stenosis caused by intraluminal bleeding tumors.

Therein, we aimed to develop a ventilation technique suitable for selected interventional rigid bronchoscopies in an attempt to address the aforementioned issues related to the current RB methodologies.

## 2. Patients and Methods

As of 2008, 782 patients with airway stenosis of different etiologies underwent interventional RB and reopening of airway lumen under general anesthesia. In 36 (4.6%) of these patients with critical central airway obstruction, we performed a novel ventilation technique using a small ventilation tube, parallel to the rigid bronchoscope (Storz Universal Rigid Bronchoscope, 8.5 mm ID, 430 mm length, Karl Storz, Tuttlingen, Germany), for anesthetic management.

We used the tracheal tube supplied in the kit of the Fantoni Translaryngeal Tracheostomy (DAR™ TLT. Trans-Laryngeal Tracheostomy KIT, Fantoni method. Covidien, 710 Medtronic Parkway, Minneapolis, MN, USA). Ventilation management was ensured using a preliminary tracheal intubation with the Fantoni Orotracheal Tube (FOT), and then placing the rigid bronchoscope parallel to the endotracheal tube. The latter was finally placed in the correct position under endoscopic control, thereby excluding use of the rigid bronchoscope as a ventilation tool, at least during tumor debulking maneuvers.

We analyzed the patients’ charts focusing on the following: radiological and endoscopic data, reports of the anesthesiologist and surgeon, and complications and short-term results. Long-term results were not evaluated considering the palliative objective of this procedure.

The outcome was deemed “satisfactory” after a complete reopening of the airway, “fairly good” after partial reopening and “poor” if inadequate palliation was achieved. Unfortunately, considering the goal of the procedure and the single procedural steps, we could not find any objective measurement to report on the results other than the surgeon’s satisfaction. In fact, the operator’s sense is currently the only tool to guide each phase of operative bronchoscopy and stenting. Moreover, the subjective improvement of respiratory symptomatology was also considered in the evaluation of the effectiveness of the procedure.

Trial registration CER Umbria, identifier 3703/19.

### Technique

The patient was placed in the traditional supine position for rigid bronchoscopy. After preoxygenation at 100% FiO_2_ with a ventilation mask for at least three minutes, anesthesia was induced via intravenous propofol. Propofol is administered with a bolus of 2 mg/kg; 0.02 to 0.5 mg/kg/minute remifentanil or 2 to 4 mg/kg fentanyl during maintenance of spontaneous or hand-assisted ventilation. Tracheal intubation was carried out using the FOT (4 mm I.D., 5.3 mm O.D., 40 cm length, large-volume/low-pressure cuff). The tracheal tube was temporarily left in a position proximal to the tumor. The operation was started by a direct introduction of the rigid bronchoscope (Storz Universal Rigid Bronchoscope, 8.5 mm ID, 430 mm length, Karl Storz, Tuttlingen, Germany) parallel to the FOT, without the use of a laryngoscope [9].

**Figure 1:**
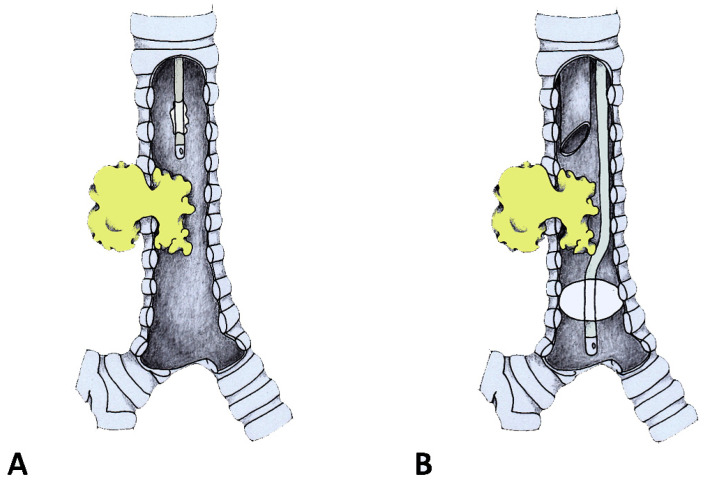
The FOT was placed above the stenosis. (**A**) The rigid scope was inserted. With this view, the FOT was gently pushed below the stenosis and then cuffed. (**B**) At that moment, the airway tract above the cuff was excluded from the one below.

Management of tracheal and carinal obstructions required different techniques. For tracheal obstructions, the FOT position was adjusted using a monitor view, passing the tip and the cuff beyond the stenosis. The cuff was then inflated so that the respiratory system below the cuff was excluded from the operative field (Figure 1). From this moment on, anesthesia was maintained with inhalation agents, and neuromuscular blockade was possible and suggested. Only after the cuff was inflated and the airway below the tube was excluded was the end-tidal capnography recorded and confirmed. A deep plane of anesthesia was continued with sevoflurane 1/2%. During the entire procedure, all the vital parameters were continuously monitored. The airway lumen reopening was accomplished using the rigid bronchoscope for operative purposes only. Once blood, secretions and tissue fragments were suctioned out, the FOT was pulled back following cuff deflation. The rigid bronchoscope could then be pushed below the stenosis, the FOT could be removed and the rigid bronchoscope resumed the role of a ventilatory channel alongside the operative one. In cases where a neuromuscular block was used, intravenous 2 to 8 mg/kg sugammadex was administered to stop it.

**Figure 2:**
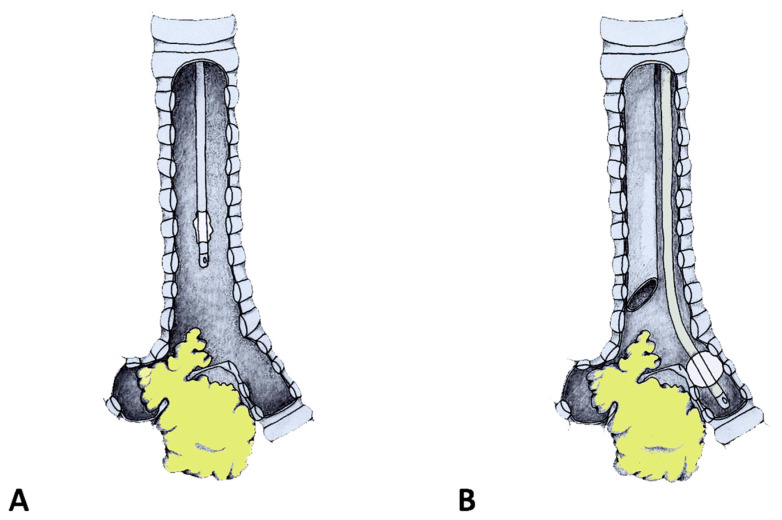
The same procedure as that shown in Figure 1A was performed. (**A**) Once the view showed the carinal obstruction and the tip of the FOT, the FOT was pushed toward the unobstructed site and the cuff was inflated at the level of the main patent bronchus. (**B**) At that moment, the healthy lung was excluded from the rest of the airway.

**Figure 3:**
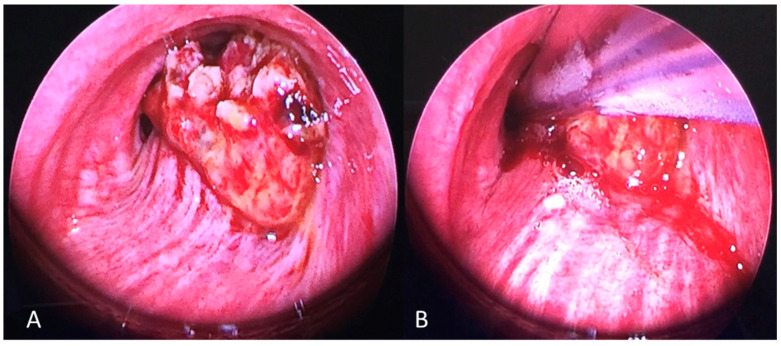
The figure shows a mid-trachea obstructive tumor leaving a very small airway lumen. (**A**) The FOT tube was passed already and cuffed below the stenosis. The upper airway was excluded from the distal airway below the cuff. The rigid bronchoscope was placed, and the operative phase could be started (**B**).

For the management of carinal obstructions, the FOT was positioned under endoscopic control into the unobstructed main bronchus, which was directly ventilated and isolated by the tube cuff (Figure 2). The rigid bronchoscope could thus be inserted into the contralateral bronchial tree avoiding ventilatory troubles and contralateral blood flooding. Intraoperative images are reported in Figure 3.

## 3. Results

Twenty-seven procedures (75%) were performed urgently. The ASA classes, as determined preoperatively, were as followed: ASA III (21 patients, 58.3%) or ASA IV (15 patients, 41.6%). Our ventilation technique was scheduled on the basis of both a preoperative flexible bronchoscopy and CT scanning. The patients had inoperable, highly symptomatic tumors of the trachea, with near-total respiratory luminal obstruction (13) or tumors of the main carina with total obstruction of one main bronchus, with or without a partial contralateral involvement (23). In the subgroup of carinal tumors, 12/23 patients received an endoscopic procedure to reopen the obstructed right main bronchus, 6/23 the left main bronchus and 4/23 had a bilateral involvement. The clinical and histological characteristics and operative details are displayed in Table 1.

The small ventilation tube passage beyond the stenosis was uneventful for all patients. In two patients with extremely severe tracheal obstruction, preliminary lumen dilatation was performed with 32Fr Chevalier Jackson bougies, which were introduced through the rigid bronchoscope. This bougie diameter was sufficient to achieve the necessary space to pass the FOT that otherwise could have been harmful. Intubation beyond the obstruction was then achieved on the guide of a thin airway exchange catheter. This step did not produce significant bleeding, impairing the subsequent steps of the procedure. In patients with carinal tumors, a 2.8 mm outer diameter fiber optic bronchoscope, introduced into the FOT, was required to appropriately place the tube into the left main bronchus. After the FOT was cuffed, the ventilation system recorded a completely sealed respiratory volume in all cases.

Ventilation and airway controls were safely managed throughout the entire surgical procedure, despite severe preoperative airway obstructions and emergency conditions. Oxygen saturation and the level of carbon dioxide fluctuated within safe ranges in all patients. Intraoperative records showed that blood pressure was maintained within the normal range in all patients during the course of the procedure. When the FOT was placed and cuffed, O_2_ saturation was easily managed and desaturation never occurred. Maintenance of anesthesia was easier than traditional rigid bronchoscopy, in which the space was operative and for ventilation at the same time. End-tidal carbon dioxide (ETCO_2_) monitoring was possible during the whole operation while the FOT was in place. Airway reopening was achieved in a single interventional session in all cases, mostly by mechanical debulking. A low-power pulsed Nd:YAG laser (20–25 W) was delivered on the surface of highly vascularized tumors before the “coring out” procedure. No endobronchial fire or explosion during the Nd:YAG laser treatment was reported, regardless of the fraction of the inspired oxygen (FiO_2_) administered [9]. In one patient, the FOT cuff was torn by the laser beam; this event did not reoccur in other patients after instilling a few millimeters of a saline solution above the cuff. No serious hemorrhages were reported in this series. Bleeding was managed using adrenaline plugging and/or cold saline solution instillation; in no case did the blood flood the distal trachea or the contralateral bronchial tree.

After the endoscopic airway lumen reopening, 18 patients received respiratory stents, which were implanted during the same operation. Sixteen Dumon stents were positioned into the trachea (3), right main bronchus (9) and left main bronchus (4). Two patients received a bifurcated silicone stent at the carinal level (Frietag Dynamic stent). The Dumon bronchial stents were positioned through the rigid bronchoscope, without removing the FOT.

A satisfactory outcome was obtained in all patients, with total resolution of symptomatology in 44.4% of patients (16) and partial resolution of symptomatology in 55.5% of patients (20). None of the patients complained of dyspnea at rest after the procedure. From a surgical point of view, in patients with carinal involvement, the outcome was satisfactory in 15 cases (65.2%), fairly good in 5 (21.7%) and poor in 3 (13%). All patients were extubated at the end of the procedure, even though four patients had to be mechanically ventilated for less than one hour with a larger orotracheal tube or with a laryngeal mask airway [9] for mild/moderate hypercarbia. No inpatient hospital deaths were reported.

## 4. Discussion

There are currently no available guidelines regarding anesthetic management during RB [10]. Nonetheless, total intravenous anesthesia is usually preferred for interventional RB as the use of anesthetic gases can be hampered by leaks in the breathing circuit through and around the bronchoscope. Several ventilation methods are also available, with their choice depending on the experience/preference of surgeon or anesthesiologist, type of airway stenosis and endoscopic technique employed. The two generally used ventilation methods for interventional rigid bronchoscopy are Spontaneous assisted ventilation and High-frequency jet ventilation (HFJV) [11,12].

Spontaneously assisted ventilation is a simple method, which preferably entails a totally intravenous anesthetic technique; it aims to maintain spontaneous ventilation with supplemental oxygen and brief assistance via bag ventilation attached to the rigid bronchoscope during the apnea and oxyhemoglobin desaturation periods. Several disadvantages have been associated with this method: bag ventilation must be interrupted while suctioning and during the entire interventional phase of the procedure, often resulting in large fluctuations of oxygen saturation. Additionally, air leakage of the breathing circuit can make the airway control unsatisfactory and does not allow for ETCO_2_ and FiO_2_ monitoring.

HFJV requires the use of a narrow injector catheter, which can easily pass through all kind of stenoses, allowing for uninterrupted ventilation [13]. However, HFJV is considered unsatisfactory for some airway stenosis requiring endoscopic palliation, and might be contraindicated when expiration is compromised [14].

Regardless of the method of ventilation adopted, airway control and ventilation management are indeed complicated in patients with life-threatening airway obstruction caused by highly vascularized tumors of the trachea. The intraluminal tumor may severely reduce the respiratory space so that distal airway control cannot be achieved through the proximally placed rigid bronchoscope, thus making spontaneously assisted ventilation unfeasible. Furthermore, a small amount of blood can heavily affect the ventilation management, as it can lead to respiratory failure with cardiovascular collapse [15]. Hence, blood dripping from highly vascularized airway tumors beyond the stenosis often cannot be cleared, causing additional obstacle to ventilation. This event represents a potential trigger for ventilation impairment and respiratory failure. Under such conditions, HFJV is certainly less hazardous than spontaneously assisted ventilation because the injector catheter can enable oxygenation. However, HFJV, being an open-breathing system, does not require an airtight seal, but it can be hazardous if the gas egress pathway is not ensured [13]. Under this circumstance, HFJV might increase the risk of barotrauma, especially in the case of tight tracheal stenosis.

Carinal tumors obstructing one of the main bronchi and protruding toward the opposite side are difficult to manage in rigid bronchoscopy; the patent bronchus, which is not intubated by the rigid bronchoscope, can be inefficiently ventilated, forcing the surgeon to periodically draw back the bronchoscope above the carina, thus interrupting the procedure. Here, anesthetic management is highly demanding whenever bloody secretions inundate the unobstructed contralateral bronchus.

After our first report on the use of the FOT tube during rigid bronchoscopy for management of central airway tumors [9], other devices have been described to ensure one-lung ventilation during challenging RB maneuvers [16,17]. To determine which one of these devices could be more useful in different settings is far beyond the purposes of this study. Our technique uses a single tube (FOT) for all stenosis types. Its diameter is very small, and the tube can even be passed through a subtotal obstruction (in the case of hindered passages, a thin airway exchange catheter can be used instead) [18]. On the other hand, in neoplastic tracheal obstructions, a thin space to pass a small catheter is always available, even in an apparently complete obstruction [19]. FOT is 40 cm long and may be easily placed beyond the carinal level, if needed. Moreover, its cuff is large in diameter and very short in length, therein permitting air sealing either at the tracheal or bronchial levels. The FOT tip is smooth and short (just 15 mm long), allowing for it to be positioned at either tracheal or bronchial levels. The positioning of the rigid scope has never been limited by the concomitant presence of the FOT. The rigid bronchoscope was fitted in each patient of the reported series, with no glottic, laryngeal or vocal cord injury occurring during the procedures. Although some obvious limitations are related to this instrumental setting, the choice of a bronchoscope smaller than the 8.5 one was never mandatory.

There are few limitations of the study. This is a descriptive study based on a retrospective review of patients undergoing palliative treatment in which objective data are difficult to find regarding the size, length and volume of the debulking procedure. Moreover, the paper is not comparative, and it reports on a series of patients belonging to a single institution over a long period of time. The case series analysis reports on palliation of respiratory impairment through airway lumen restoration without any standardized outcome or intention. Further studies are needed to confirm these promising results. Comparative trials with standard technique control could be definitive.

Besides the study limitations, this technique appears to be advantageous for the following reasons:

- In our experience, despite the very high-risk procedure, the anesthetic and surgical management were uneventful in all patients. Although the FOT has a small diameter, which could induce a higher resistance, thus requiring greater airway pressures, airway management has been consistently satisfactory with regard to respiratory parameters, without problems in controlling ventilation. Since the flux of gas was modified when the oro-tracheal tube was 5.0, the volume and scheme of ventilation had to be adjusted. Good ventilation control was reported for both tracheal and carinal tumors. At the tracheal level, the FOT was able to bypass any obstruction and, due to its wide cuff, it ensured air tightness. At the carinal level, intubation of the unobstructed main bronchus with the long FOT allowed for the insertion of the rigid endoscope into the contralateral diseased main bronchus, without ventilation impairment.
- The risk associated with distal blood dripping was avoided. Distal floods of blood caused by the debulking of the tumor are possible with all the ventilation techniques currently used and can cause severe respiratory deficiency, mainly because aspiration beyond obstruction is impossible before the tracheal lumen has been reopened.
- The anesthesia of such difficult and risky procedures is simplified: the levels of FiO_2_ and ETCO_2_ can be constantly monitored, neuromuscular blocking is possible and even anesthetic inhalation techniques are available, if necessary. Furthermore, FiO_2_ can be set to the desired level even when using the laser.
- The endoscopic procedure of airway reopening is accelerated as the intervention does not require pauses to ventilate the patient.

The only disadvantage is that the technique is more expensive than a common orotracheal tube. In fact, the FOT is not commercially available alone and an entire kit of the Fantoni TLT must be wasted to use only the tube. The higher cost of the Fantoni TLT technique is a significant issue, especially in regard to the limited number of cases (4.6% in this series) in which this technique might be indicated. Thus, considering the lack of availability of the FOT as an individual component may hinder innovation and customization. A Phycon tube (Fuji Systems Corporation, Fukushima, Japan) or an adult microlaringoscopy 5.0 tube could be alternatives to explore for this indication, although the diameter is slightly larger and the cuff does not have the same characteristics. We have never adopted these options to date, so no technical comment is possible. Finally, even though maneuverability of the rigid bronchoscope is slightly reduced because of the simultaneous endotracheal presence of the FOT, we did not find technical hindrance during the operative phase of the RB.

## 5. Conclusions

Our experience shows the feasibility and safety of this new method of airway management during RB for neoplastic tracheal and/or carinal stenosis. This technique allows for continuous ventilation control, preventing some of the common technical issues of rigid bronchoscopy, facilitating both the anesthesiologist and surgeon. The described technique is not revolutionary, but represents a potentially valid alternative option for the management of central airway tumors with palliative intent.

## Figures and Tables

**Table 1:** Demographics, clinical and surgical information.

	Tracheal Stenosis (13)	Carinal Stenosis (23)
**Clinical Characteristic**		
Male	8 (61.5%)	15 (65.2%)
Female	5 (38.5%)	8 (34.8%)
Dyspnea	13 (100%)	23 (100%)
Irrepressible cough	10 (76.9%)	18 (78.3%)
Hemoptysis	1 (7.7%)	4 (17.4%)
**Histological characteristic**		
Adenocarcinoma	3 (23.1%)	6 (26.1%)
Squamous cell carcinoma	7 (53.8%)	12 (52.2%)
Neuroendocrine large cell carcinoma	0	1 (4.3%)
Small cell carcinoma	0	1 (4.3%)
Lymphoma B	1 (7.7%)	1 (4.3%)
Undifferentiated large cell carcinoma	1 (7.7%)	1 (4.3%)
Metastasis from enteric adenocarcinoma	0	1 (4.3%)
Esophageal squamous cell carcinoma	1 (7.7%)	0
**Surgical characteristic**		
Urgent procedure	7 (53.8%)	20 (86.9%)
Need of preliminary airway reopening	2 (15.4%)	22 (95.6%)
Airway stenting	3 (23.1%)	15 (65.2%)
Perioperative mortality	0	0

## Data Availability

The data presented in this study are available on request from the corresponding author. The data are not publicly available due to patient privacy.
